# Adenovirus-associated anti-miRNA-214 regulates bone metabolism and prevents local osteoporosis in rats

**DOI:** 10.3389/fbioe.2023.1164252

**Published:** 2023-05-12

**Authors:** Cheng Wang, Peng Wang, Feng Li, Yang Li, Minwei Zhao, Hui Feng, Haoye Meng, Junyang Li, Peng Shi, Jiang Peng, Hua Tian

**Affiliations:** ^1^ Peking University Third Hospital, Department of Orthopaedics, Engineering Research Center of Bone and Joint Precision Medicine, Ministry of Education, Beijing Key Laboratory of Spinal Disease Research, Beijing, China; ^2^ Institute of Orthopaedics, Beijing Key Laboratory of Regenerative Medicine in Orthopedics, Key Laboratory of Musculoskeletal Trauma & War Injuries PLA, The Fourth Medical Center of the General Hospital of People's Liberation Army, Beijing, China; ^3^ Department of Electronic Engineering, Ocean University of China, Qingdao, China; ^4^ Centre for Robotics and Automation, Shenzhen Research Institute of City University of Hong Kong, Shenzhen, China

**Keywords:** osteoporosis, bone metabolism, miRNA-214, osteoblast activity, osteoclast activity

## Abstract

**Objective:** We investigated the expression of miRNA-214 in human osteoporotic bone tissue and tested the utility of adeno-associated virus (AAV) expressing a miRNA-214 inhibitor in terms of preventing local osteoporosis of the femoral condyle in a rat model of osteoporosis.

**Methods:** (1) Femoral heads of patients who underwent hip replacements at our hospital because of femoral neck fractures were collected and divided into osteoporosis and non-osteoporosis groups based on preoperative bone mineral density data. MiRNA-214 expression was detected in bone tissues exhibiting obvious bone microstructural changes in the two groups. (2) A total of 144 SD female rats were divided into four groups: the Control, Model, Negative control (Model + AAV), and Experimental (Model + anti-miRNA-214) groups. AAV-anti-miRNA-214 was injected locally into the rat femoral condyles; we explored whether this prevented or treated local osteoporosis.

**Results:** (1) MiRNA-214 expression in the human femoral head was significantly increased in the osteoporosis group. (2) Compared to the Model and Model + AAV groups, the bone mineral density (BMD) and femoral condyle bone volume/tissue volume (BV/TV) ratio in the Model + anti-miRNA-214 group were significantly higher; in addition, the number (TB.N) and thickness (TB.Th) of the trabecular bones were increased (all *p* < 0.05). MiRNA-214 expression in the femoral condyles of the Model + anti-miRNA-214 group was significantly higher than that in the other groups. The expression levels of the osteogenesis-related genes *Alp*, *Bglap*, and *Col1α1* increased, while those of the osteoclast-related genes *NFATc1*, *Acp5*, *Ctsk*, *Mmp9*, and *Clcn7* decreased.

**Conclusion:** AAV-anti-miRNA-214 promoted osteoblast activity and inhibited osteoclast activity in the femoral condyles of osteoporotic rats, improving bone metabolism and slowing osteoporosis progression.

## 1 Introduction

Osteoporosis is a common orthopedic disease of middle-aged and elderly individuals. The common clinical features include systemic bone mass reduction and destruction of the bone microstructure. The incidence of osteoporosis has increased in recent years (Johnston and Dagar, 2020). However, osteoporosis awareness is low. Moreover, disease onset is insidious, and in early stages, patients are often asymptomatic, or the symptoms are very mild. Thus, the condition is often not detected. Serious osteoporosis can cause back and leg pain, spinal deformities, and even fractures that seriously compromise the quality of life, shorten life expectancy, and impose heavy burdens on societies and families (LeBoff et al., 2022). There is an urgent need to reduce the incidence of osteoporosis, inhibit bone mass reductions, prevent declines in mechanical strength, and reduce the fracture rate (Arceo-Mendoza and Camacho, 2021). Osteoporosis is a complex disease. It is believed that an imbalance between bone formation and resorption is the principal cause. Thus, if bone metabolism could be regulated via human intervention, natural osteoporosis progression might be prevented and the condition adequately treated.

We previously found that the expression of miRNA-214 was significantly higher in a mouse model of bone loss induced by tail suspension than in the control group, and it correlated negatively with the bone expression levels of ALP and OCN (Sun et al., 2016). Microarray analysis showed that in mature osteoclasts, the expression of miRNA-214 was most significantly affected. We confirmed that miRNA-214 targeted ATF4 to inhibit osteoblast activity and PTEN/PI3K/Akt to promote osteoclast activity (Zhao et al., 2015). Might miRNA-214 serve as an early diagnostic marker of osteoporosis and as a useful therapeutic target?

We collected femoral head samples from patients with femoral neck fractures after they underwent hip replacements and divided the samples into osteoporosis and non-osteoporosis groups by reference to preoperative dual-energy X-ray bone density screening. We measured miRNA-214 expression levels in the bone tissue. We also sought a drug that effectively (bidirectionally) regulated bone metabolism. We tested anti-miRNA-214 (an inhibitor of miRNA-214), which promotes osteogenesis and inhibits osteoclast action. We used this model to treat rat osteoporosis and explored the mechanism in play. We sought new directions toward the treatment of local osteoporosis.

## 2 Methods

### 2.1 Clinical sample collection and analysis

Twelve femoral head samples from twelve patients with femoral neck fractures undergoing hip replacements were collected.

Patients subjected to dual-energy X-ray tests prior to hip replacement (T ≥ −1.0 normal, −1.0 > T > −2.5 osteopenia, and T ≤ −2.5 osteoporosis) were included. To enhance rigor and reflect the real-world situation, patients with T values ≤ −3 formed the osteoporosis group, and those with T values ≥ −1.5 formed the non-osteoporosis group. There were six patients in each group, aged 65–75 years, without systemic or local infection. There were no hip diseases, such as hip osteoarthritis, osteonecrosis of the femoral head, or developmental dysplasia of the hip.

The exclusion criteria were any tumor, diabetes, hyperthyroidism, severe liver or kidney disease, and/or severe rheumatic immune disease. Patients with excessive smoking, alcohol, substance abuse, or mental illness were excluded.

### 2.2 Detection of miRNA-214 in the clinical samples

We subjected the clinical samples to high-resolution microcomputed tomography (CT) and then measured the miRNA-214 levels in the internal bone tissues. We sought a correlation between a change in the bone microstructure and the local miRNA-214 expression level.

### 2.3 Preparation and evaluation of adeno-associated virus (AAV)-anti-miRNA-214

The inhibitor of miRNA-214 (anti-miRNA-214) lost activity rapidly in aqueous solutions. Systemic application is compromised by pH and humidity, and the material may exert unknown effects on other organs and systems. Local puncture/fractional administration does not ensure the accuracy of release. AAV has become the most promising gene therapy tool known. The safety profile is good, and its expression persists in the long term (Li and Samulski, 2020; Large et al., 2021). The type of AAV used was 9, which is called AAV9, and the concentration was 10^13^ vg/ml. Based on our previous research, we synthesized AAV-anti-miRNA-214-expressing green fluorescent protein (GFP) to facilitate tracking. We previously synthesized AAV-anti-miRNA-214 and functionally evaluated the material at the cellular level. We found that 30 µL is an excellent measurement (Wang et al., 2019).

### 2.4 The animal model of osteoporosis

A total of 144 3-month-old female SD rats were randomly divided into four groups: 1. Control, 2. Model, 3. Model + Negative control (Model + AAV), and 4. Model + AAV-anti-miRNA-214 (Model + anti-miRNA-214). The Control group received no treatment, and the Model group underwent only bilateral ovariectomy (OVX). In the Model + AAV group, 30 µL of an AAV suspension was injected into the right lateral femoral condyle at the time of the bilateral OVX; in the Model + AAV-anti-miRNA-214 group, 30 µl of an AAV-anti-miRNA-214 suspension was injected into the right lateral femoral condyle at the time of the OVX. The animal study was reviewed and approved by the Peking University Third Hospital Medical Science Research Ethics Committee.

Osteoporosis was triggered by the OVX. After the rats were anesthetized, the skin was prepared, and the operative area was disinfected with iodophor and a sterile towel spread. With each rat prone, the skin and muscle were dissected longitudinally 1 cm from the right side of the spine and 1 cm below the costal margin. After blunt dissection, pink cauliflower-shaped ovaries were evident when the cellulite was removed. The ovaries and oviducts were excised, ligated near the uterus, and the wounds were closed.

Drug injection: The skin of the right knee was cut and exposed layer-by-layer to the bone surface of the lateral femoral condyle. An empty syringe (500 µl) was used to puncture the bone. This was replaced by a brand-new syringe, and 30 µl of an AAV or AAV-anti-miRNA-214 suspension was slowly injected. The syringe was held in place for 5 min and then removed. Then, the wound was closed. The right femoral condyles were collected 4, 8 weeks after the OVX and subjected to micro-CT, fluorescence microscopy, and pathological evaluation. MiRNA-214 expression and osteoblast and osteoclast activity levels were assayed in the 8-week samples.

### 2.5 Bone microstructural analysis

#### 2.5.1 Micro-CT, X-ray imaging, and bone morphometric analysis

At 4 and 8 weeks after the OVX, the right femoral condyles were harvested, subjected to X-ray imaging, and then detected by micro-CT using standard settings: resolution 27 × 27 × 27 μm, scan current 450 mA, scan voltage 80 kV, and scan time 88 min. The 8-week samples were subjected to three-dimensional reconstruction. We delineated the region of interest (ROI) within the femoral condyle. The axial surface of the epiphyseal plate served as the baseline. Overall, 50 scan layers were selected for both sides (100 in total), and the epiphyseal plate contour was delineated at 10-layer intervals. Each ROI avoided cortical bone, instead prioritizing cancellous bone. Bone morphometric analysis was performed 4 and 8 weeks after the OVX. We evaluated the BMD, bone volume/tissue volume (BV/TV), and trabecular number (Tb.N), thickness (Tb.Th), and spacing (Tb.sp).

#### 2.5.2 AAV transfection evaluation and pathological examination

At 4, 8 weeks after the OVX, the rats in each group were sacrificed via the induction of deep anesthesia. The right femoral condyles were collected, embedded in a tissue-freezing medium, cut into frozen sections, and stained with 4′,6-diamidino-2-phenylindole hydrochloride (DAPI) for 5 min. Fluorescence microscopy was employed to assess the viral transfection status.

The samples were fixed in 4% (v/v) paraformaldehyde for 2 days, decalcified with 10% (w/v) ethylene diamine tetra-acetic acid, subjected to gradient dehydration and paraffin embedding, cut into 7-μm-thick sections, and subjected to hematoxylin–eosin (HE) staining.

### 2.6 MiRNA-214 expression and osteoblast and osteoclast activities

At 8 weeks after the OVX, fresh femoral condyles were collected for PCR to measure the expression levels of miRNA-214, the osteoblast activity-related genes *Alp*, *Bglap*, and *Col1α1*, and the osteoclast activity-related genes *NFATc1*, *Acp5*, *Ctsk*, *Mmp9*, and *Clcn7*.

### 2.7 Statistical analysis

All statistical analyses were performed with SPSS ver. 20.0 statistical software; an analysis of variance was used to compare among-group data. All results are presented as the means ± standard deviations (SDs), and *p* < 0.05 was considered statistically significant.

## 3. Results

### 3.1 MiRNA-214 expression in human osteoporosis samples

**FIGURE 1:**
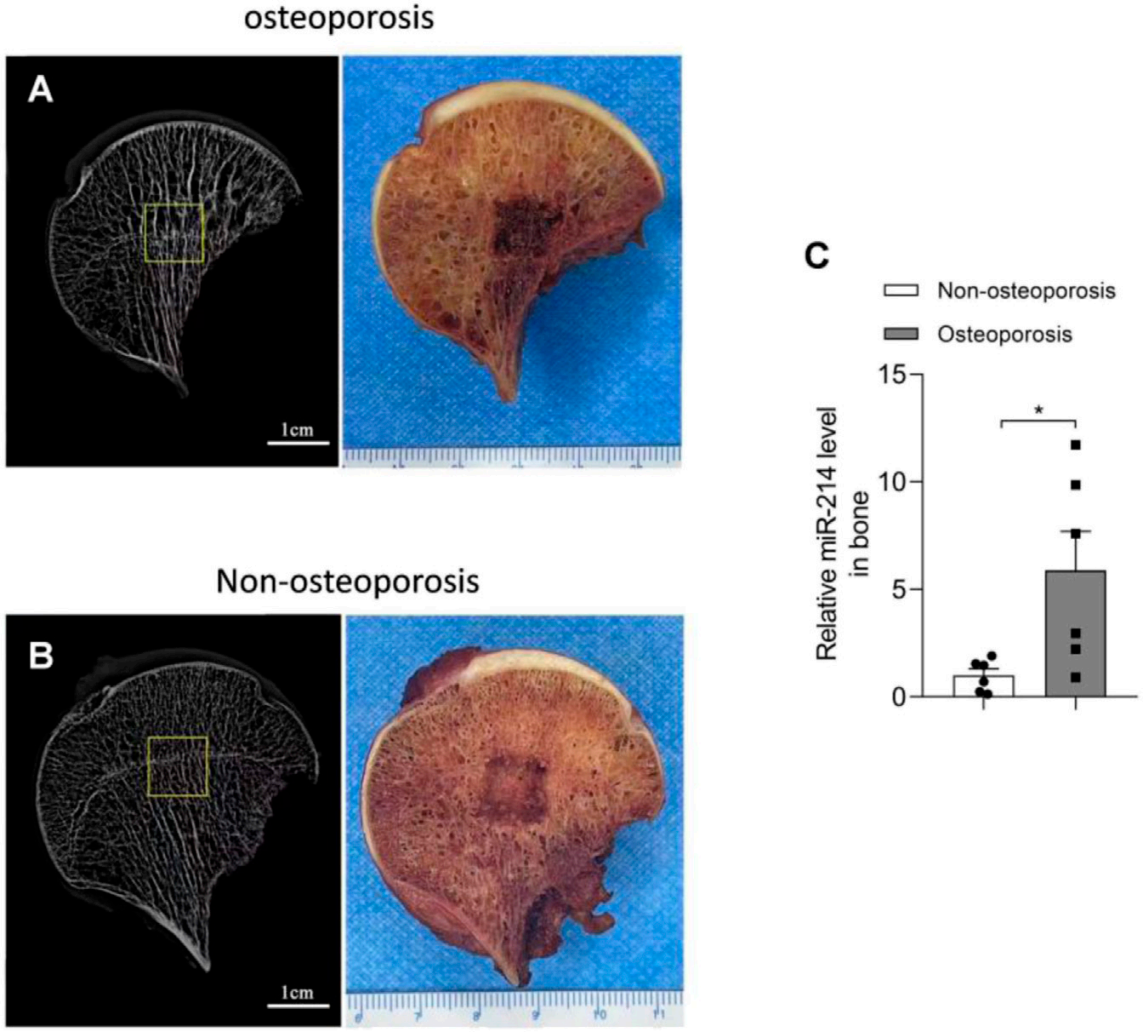
Micro-CT results and miRNA-214 expression levels in femoral head samples from both groups. **(A)** Bone microstructure of femoral head samples from the osteoporosis group and a schematic of the affected region. **(B)** Bone microstructure of femoral head samples from the osteoporosis group and a schematic of the affected region. **(C)** MiRNA-214 expression in osteoporotic tissue was significantly higher than that in non-osteoporotic tissue. For both groups, n = 6. All data are means ± SDs (*p<0.05).

We used the micro-CT data to select areas with obvious changes in bone microstructure and subjected samples from these areas to PCR. MiRNA-214 expression in the osteoporotic bone tissue was significantly increased (compared to normal tissue); the difference was significant (*p* < 0.05) (Figure 1).

### 3.2 Bone microstructure morphometry in osteoporotic rats

**FIGURE 2:**
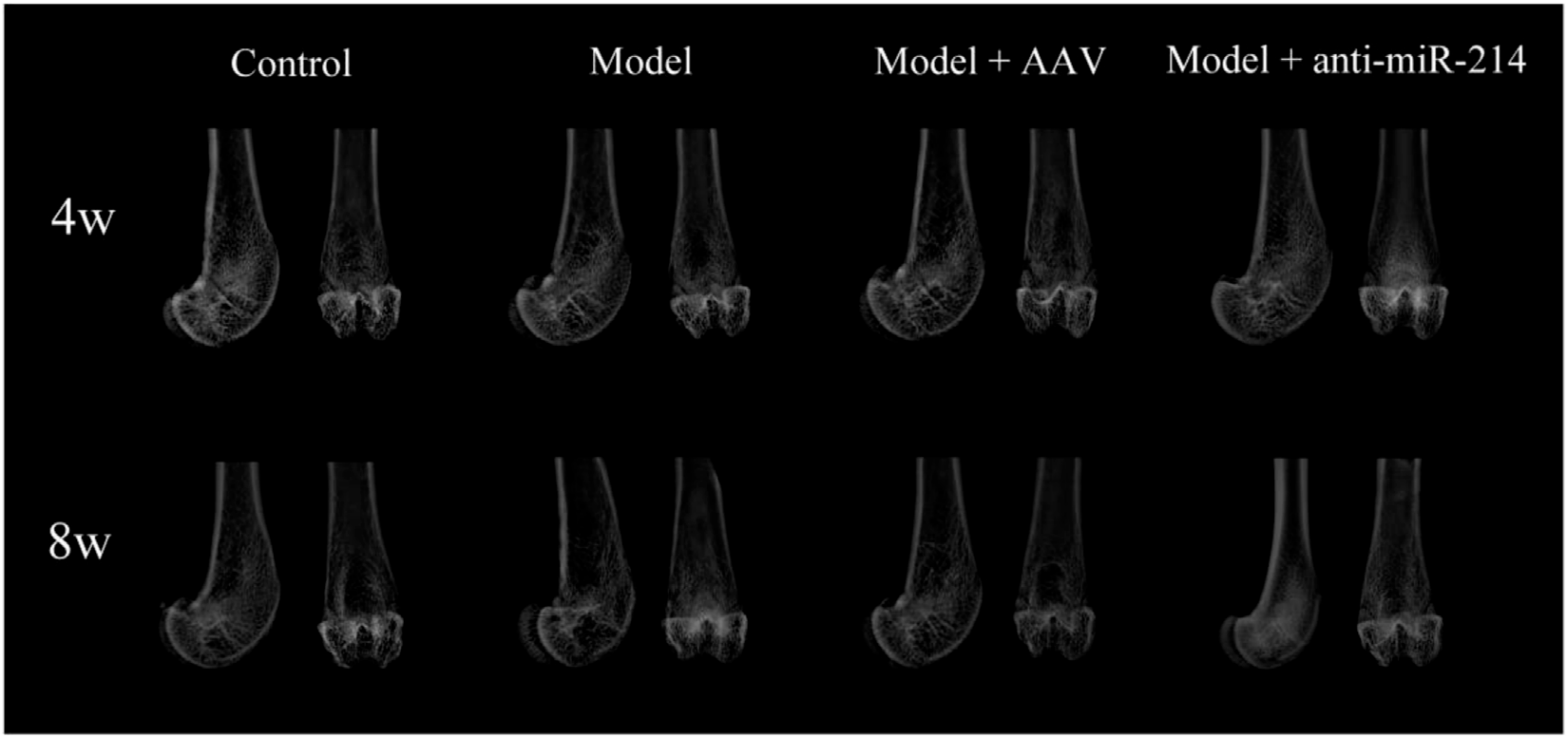
X-ray imaging performed at 4 and 8 weeks after OVX. Anteroposterior and lateral radiographs of the femoral condyles of the various groups.

The 4- and 8-week X-rays showed that the femoral condyles of the Control group exhibited uniform bone density and good structural integrity. The femoral condyle bone density decreased gradually over time in the Model and Model + AAV groups. The differences became more significant over time. However, this was not the case for the Model + anti-miRNA-214 group, and better bone mineral density was maintained in the femoral condyle (similar to the Control group) (Figure 2).

**FIGURE 3:**
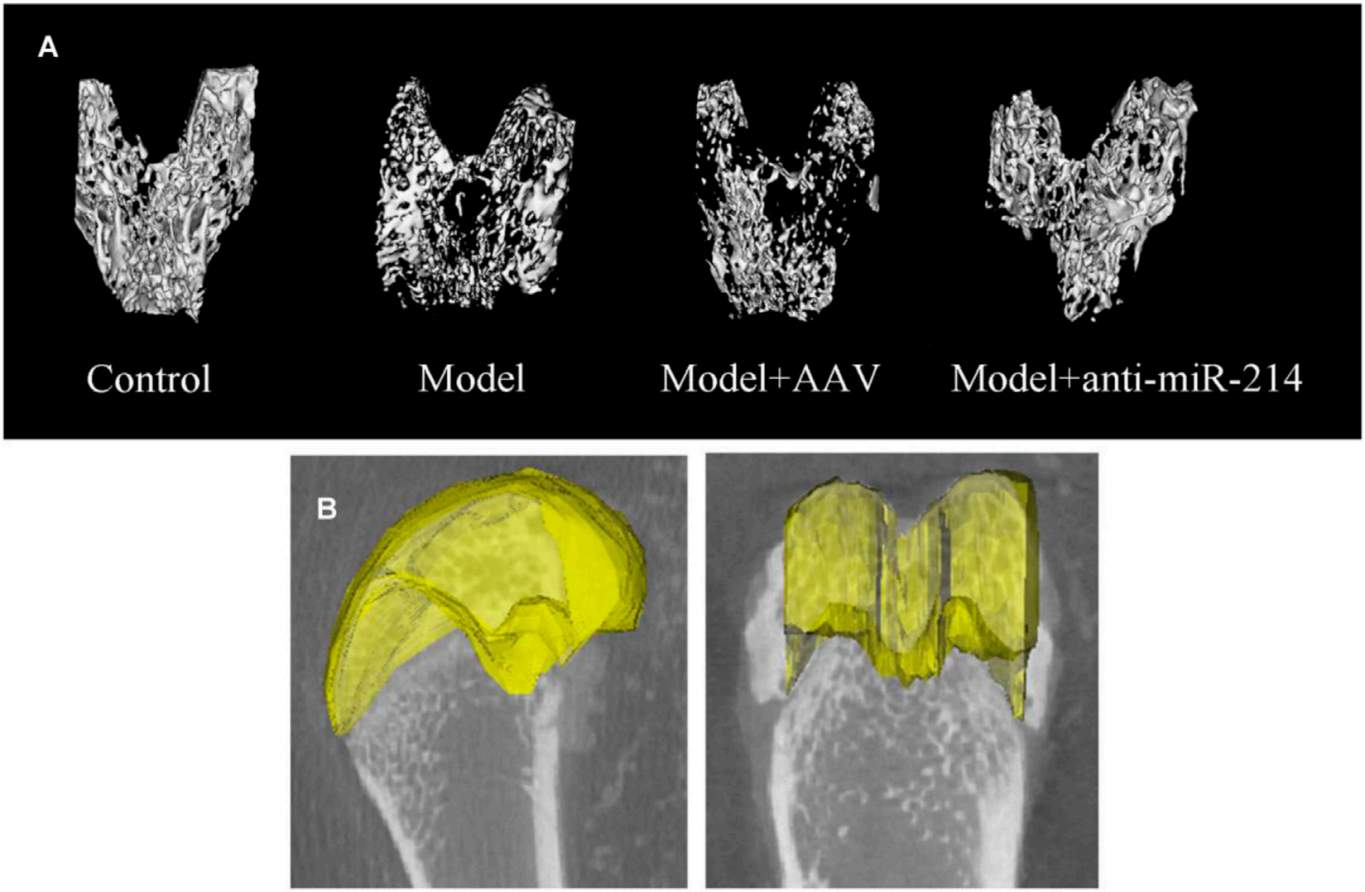
**(A)** Condylar micro-CT three-dimensional reconstructions at 8 weeks after OVX for rats in different groups. **(B)** Selection of an ROI. The axial surface of the epiphyseal plate served as the baseline. Overall, 50 scan layers were selected from either side (100 in total), and the epiphyseal plate contours were determined at 10-layer intervals.

The micro-CT three-dimensional reconstructions showed that the femoral condyle microstructures of different groups differed significantly 8 weeks after the OVX. In the Control group, the trabecular bone structure was continuous and evenly distributed. In the Model and Model + AAV groups, the bone density decreased gradually, the number of trabeculae fell, and most trabecular bone continuity was lost over time. In the Model + anti-miRNA-214 group, the high bone mineral density was preserved, and the trabecular bone structure was basically intact (without obvious osteoporosis) (Figure 3).

**FIGURE 4:**
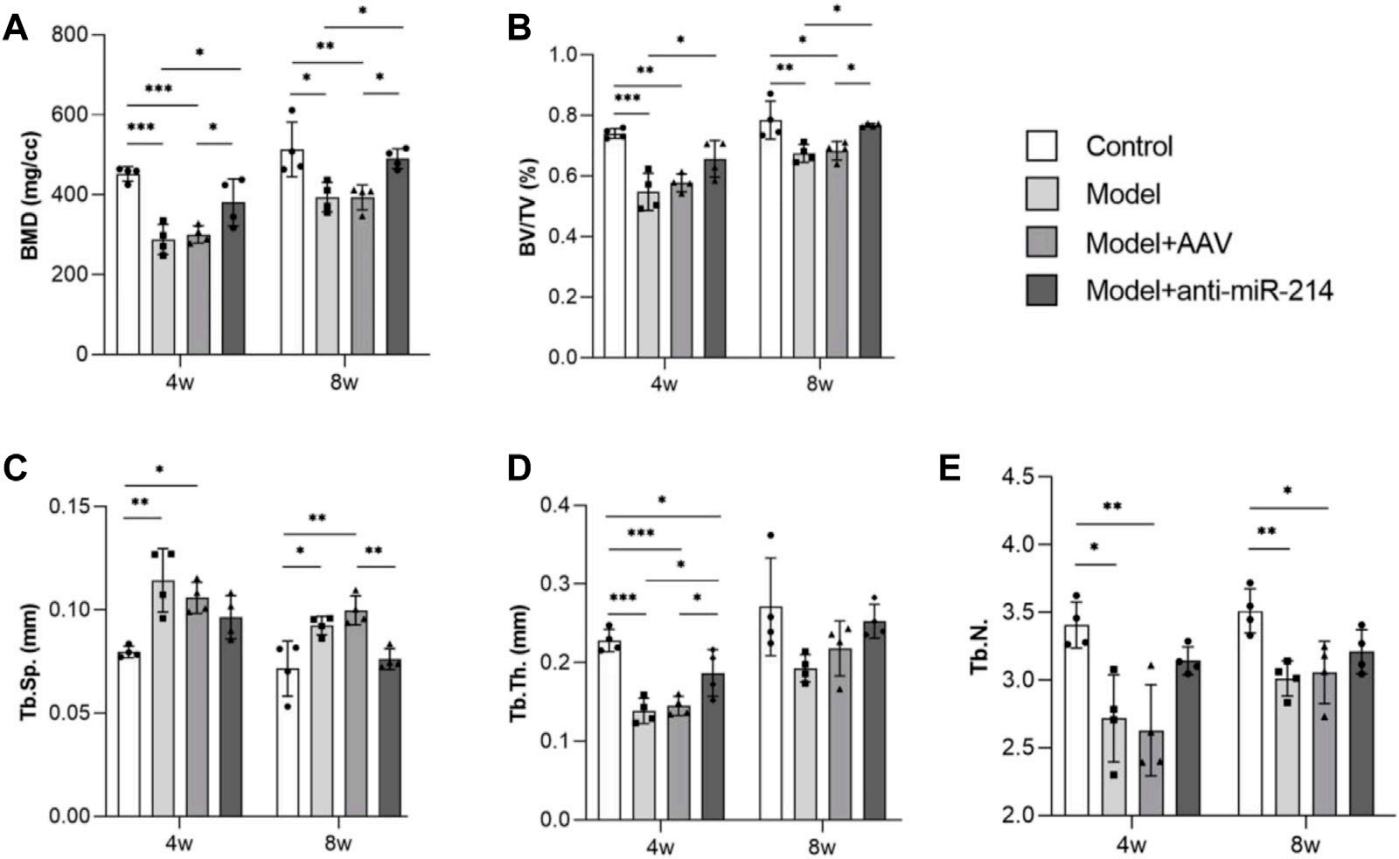
Bone morphometric analyses at different times (BMD, BV/TV, Tb.N, Tb.Th, and Tb.sp data). For each group, n = 6. All data are means ± SDs. (*p<0.05; **p<0.01; ***p<0.001)

The bone morphometry results showed that compared to what was observed in the Model + anti-miRNA-214 group, the BMD and BV/TV of the Model and Model + AAV groups were significantly decreased (both *p* < 0.05). The trabecular number and thickness were also decreased, and the spacing was increased. These differences were statistically significant (all *p* < 0.05) (Figure 4).

### 3.3 AAV transfection evaluation

**FIGURE 5:**
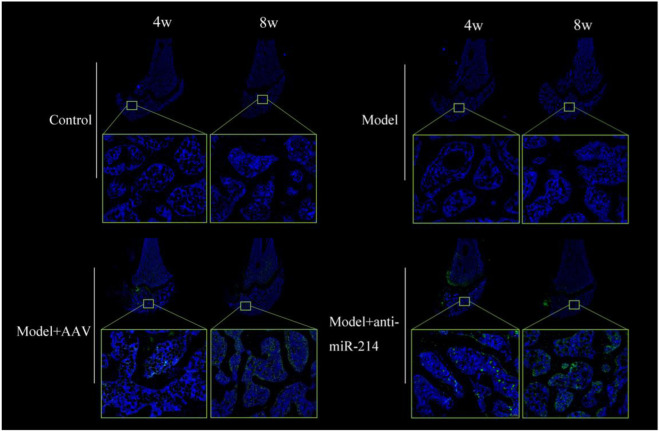
Fluorescence images of frozen femoral condyle sections at 4 and 8 weeks after OVX. Blue and green fluorescent signals were evident in the Model + AAV and Model + anti-miRNA-214 groups; the signals were stronger at 8 weeks.

The Control and Model groups showed only blue fluorescence at 4 and 8 weeks after the OVX; there was no green fluorescence. However, blue and green fluorescence was apparent in the Model + AAV and Model + anti-miRNA-214 groups; the signals were stronger at 8 weeks. Thus, the AAV virus infected the femoral condyles and replicated extensively (Figure 5).

### 3.4 Bone histological assessment

**FIGURE 6:**
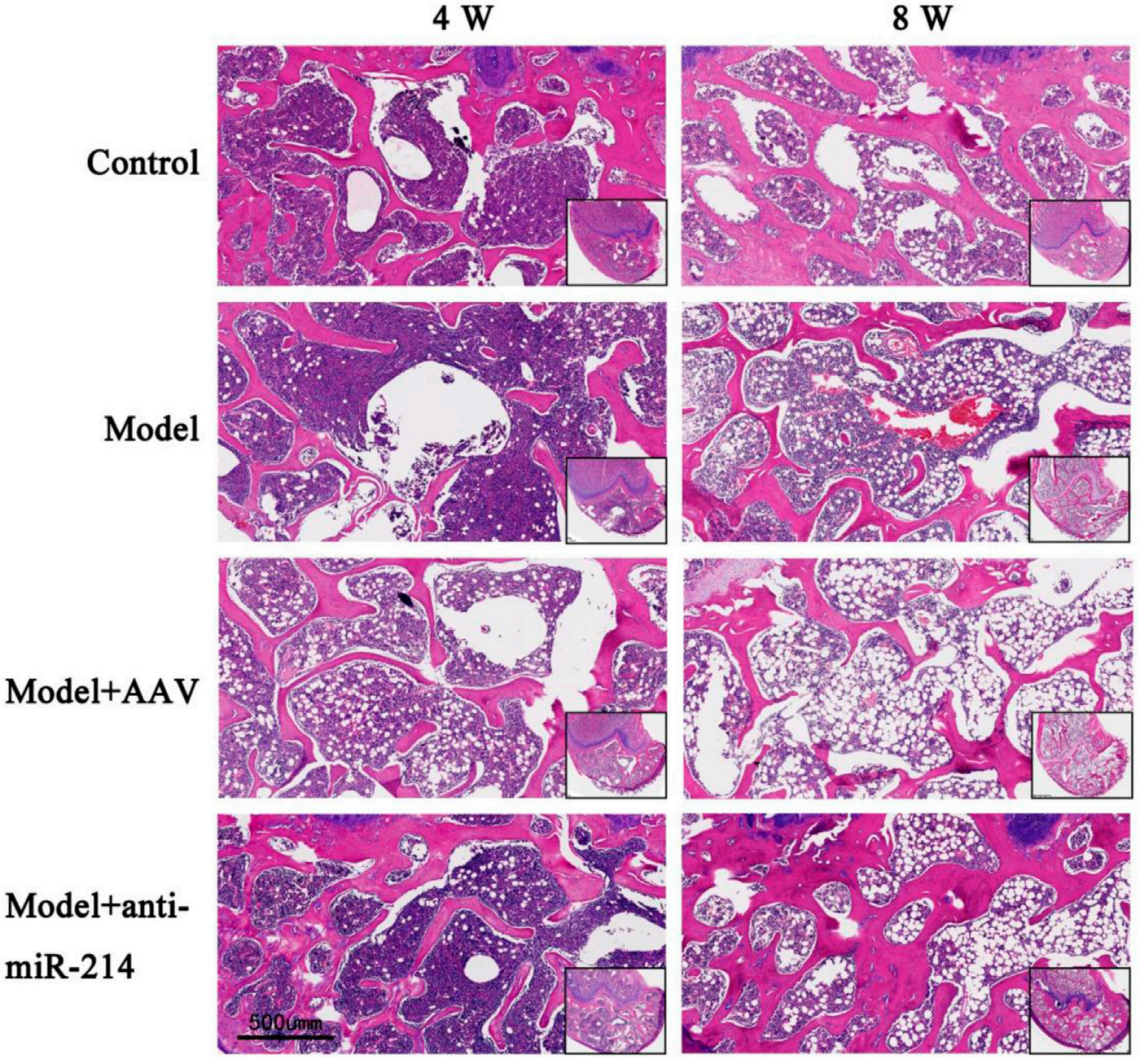
Condylar HE staining of the Control, Model, Model + AAV-AAV, and Model + AAV-anti-miRNA-214 groups 4 and 8 weeks after OVX.

The rats were sacrificed 4 and 8 weeks after the OVX, and the condylar paraffin sections were HE-stained. The bone trabeculae of the Control group were orderly and intact, and the osteocyte density was uniform. In the Model and Model + AAV groups, the bone trabecular number and density decreased, the continuities of some trabeculae were interrupted, the number of bone lacunae gradually increased, and the number of osteocytes gradually decreased over time. However, the trabeculae of the Model + anti-miRNA-214 group were orderly, and the number of trabeculae and the osteocyte density were similar to those of the Control group (Figure 6).

### 3.5 Expression of miRNA-214 and genes related to osteoblast and osteoclast activity

**FIGURE 7:**
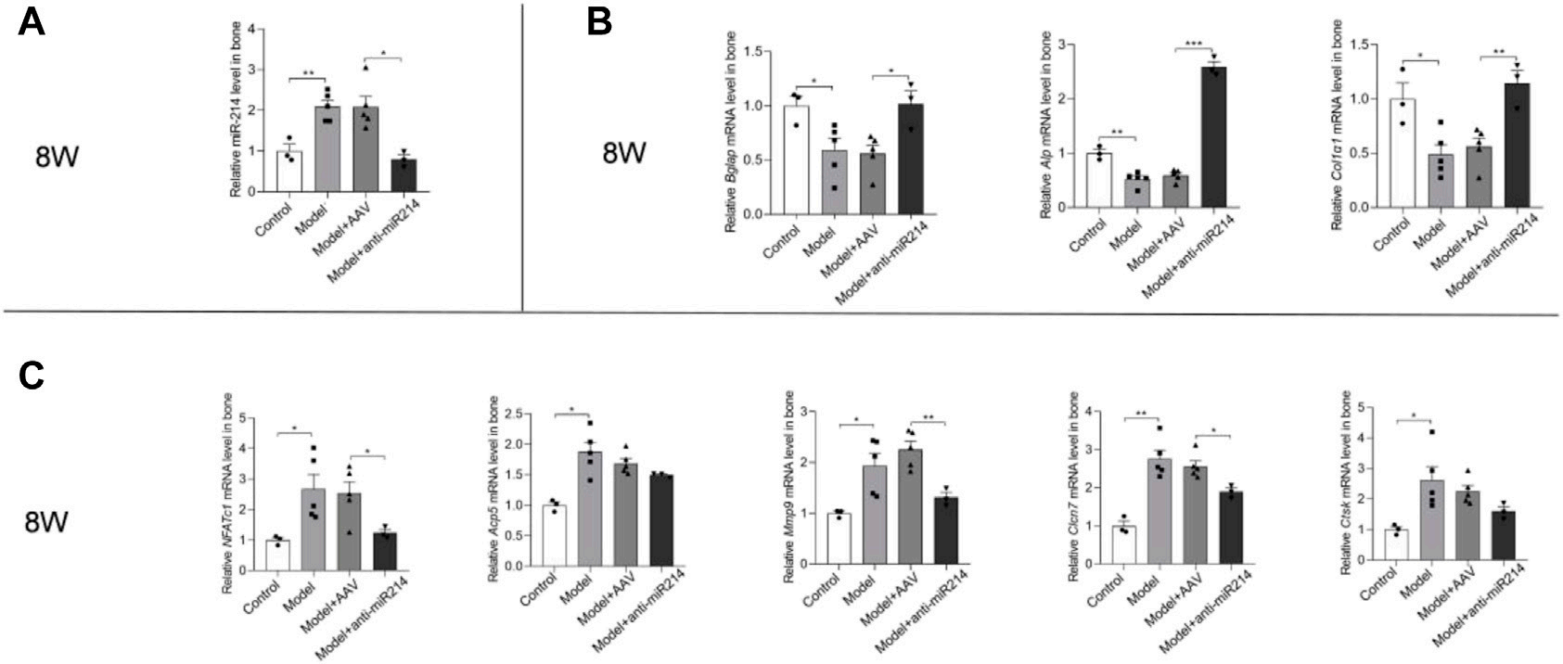
**(A)** Quantitative real-time PCR analyses of the miRNA-214 levels in rat femoral condyles 8 weeks after OVX. **(B)** Expression levels of the osteoblast-associated genes *Alp, Bglap, and Col1α1*. **(C)** Expression levels of the *NFATc1*, *Acp5*, *Ctsk*, *Mmp9*, and *Clcn7* genes. For each group, *n* = 6. All data are means ± SDs (*p<0.05; **p<0.01; ***p<0.001).

MiRNA-214 levels were detected via PCR. At 8 weeks after the OVX, the miRNA-214 levels in the Model and Model + AAV groups increased, and there was no significant difference in the expression of miRNA-214 in the Model + anti-miRNA-214 group compared with the Control group (Figure 7A). At 8 weeks after the OVX (compared to the Control group), the expression of the osteoblast activity-related genes *Alp*, *Bglap*, and *Col1α1* was significantly decreased in the Model and Model + AAV groups but not in the Model + anti-miRNA-214 group. The expression levels of the osteoclast activity-related genes *NFATc1*, *Acp5*, *Ctsk*, *Mmp9*, and *Clcn7* were significantly increased in the Model and Model + AAV groups, but the levels in the Model + anti-miRNA-214 and Control groups did not significantly differ (Figures 7B,C).

## 4 Discussion

The prevention of osteoporosis would improve bone growth and development, maintain bone quality and mass, and prevent age-related bone loss, falls, and fractures (Munoz et al., 2020). Treatments include basic lifestyle adjustments and bone health supplements. Anti-osteoporosis drugs include vitamin D3, salmon calcitonin, calcitriol, and bisphosphonates (Kanis et al., 2019). However, no drug bidirectionally and efficiently regulates osteogenic and osteoclastic processes. Moreover, some treatments have side effects (mandibular osteonecrosis and renal toxicity) (Fink et al., 2019). Thus, a new approach is needed. Osteoporosis is a systemic metabolic disease; few studies have focused on the prevention and treatment of local osteoporosis. In fact, most patients with osteoporosis do not see a doctor prior to an osteoporotic fracture. Clinically, most such fracture sites are in the hip, spine, or distal radius. If the osteoporosis screening intensity of the high-risk age group were increased, might local anti-osteoporosis treatment of sites prone to fracture be of assistance? Can we reduce the incidence of osteoporotic fractures?

MiRNAs regulate the proliferation, differentiation, and functional activity of various bone cells, playing roles from embryonic bone development to adult bone reconstruction (Kabekkodu et al., 2018; Tafrihi and Hasheminasab, 2019). Lin et al. (Lin et al., 2019) found significantly higher expression of miRNA-338 clusters in postmenopausal patients with osteoporosis than in those without osteoporosis, and they described an estrogen-dependent Runx2/Sox4/miR-338 positive feedback loop regulating osteoblast differentiation. More importantly, the inhibition of miRNA-338 expression delayed the progression of postmenopausal osteoporosis. Hu et al. (Hu et al., 2011) found that miR-2861 targeted Hdac5, a factor inhibiting the transcription of *RUNX2* (which mediates bone formation), and found that miRNA-3960 targeted Hoxa2 (a repressor of Runx2) to promote osteoblast differentiation. Zhao et al. (Zhao et al., 2021) found that miR-483-5p was upregulated and SATB2 was downregulated in clinical patients with osteoporosis. After mechanistic research, they recognized that miR-483-5p contributed to the pathogenesis of osteoporosis by inhibiting SATB2 and activating the PI3K/AKT pathway. Yu et al. (Yu et al., 2020) also conducted similar research on osteoporosis. After analyzing microarray data and careful verification, they found that patients with osteoporosis had higher miR-16-5p and lower VEGFA levels. Experiments with stem cells have shown that miR-16-5p suppresses osteogenesis by inhibiting VEGFA expression. Wang et al. (Wang et al., 2013) previously confirmed that miRNA-214 targeted ATF4 in osteoblasts, inhibiting cellular activity.

MiRNAs also play important roles in the regulation of osteoclast differentiation and function (Inoue et al., 2019; Lozano et al., 2019). The Krzeszinski team (Krzeszinski et al., 2014) found that the gene encoding transforming growth factor-β-induced factor 2, a direct target of miR-34a, played a role in promoting bone resorption. As a key osteoclast suppressor, miR-34a may serve as a therapeutic target in patients with osteoporosis and metastatic bone cancer. Sugatani et al. (Sugatani et al., 2011) found that miRNA-21 expression was regulated by various transcription factors (especially c-Fos) during RANKL-induced osteoclast differentiation. Rankl-induced c-Fos upregulated miRNA-21 and then downregulated the expression of programmed cell death protein 4 (PDCD4). This constitutes a c-Fos/miR-21/PDCD4 positive feedback loop that regulates osteoclast function. Our preliminary data show that miRNA-214 slows the inhibition of PI3K/AKT by PTEN, activates the downstream signaling pathway of osteoclasts, and enhances osteoclast activity (Zhao et al., 2015).

We previously performed a detailed analysis of femoral heads with osteonecrosis. Obvious local changes in bone metabolism were apparent. In necrotic areas, osteoclast activity was significantly enhanced, and osteoblast activity was weakened. However, the opposite result was found in sclerotic regions. Therefore, we used an AAV expressing the GFP reporter gene and an inhibitor of miRNA-214 to treat femoral head osteonecrosis in rats. The drug effectively regulated osteoblast and osteoclast activities and prevented osteonecrotic collapse (Wang et al., 2019). Here, we found that the internal bone microstructure of human osteoporotic samples exhibited obvious changes. MiRNA-214 expression was significantly upregulated in osteoporotic tissue, and this was negatively correlated with the expression of genes of osteoblast and osteoclast activities. Thus, miRNA-214 may play a regulatory role in the pathogenesis of osteoporosis.

We thus used AAV-anti-miRNA-214 to locally regulate osteogenic and osteoclastic processes; we sought to change or even reverse osteoporosis. After the OVX, condylar osteoporosis gradually developed in the Control rats, and the miRNA-214 level in the bone increased significantly, suggesting that miRNA-214 is associated with osteoporotic pathogenesis. After the OVX, condylar osteoporosis gradually developed in the rats of the Model and Model + AAV groups, and the bone expression of miRNA-214 increased significantly. Again, these findings suggest that miRNA-214 is associated with osteoporotic pathogenesis. The rats in the Model + anti-miRNA-214 group received AAV-anti-miRNA-214 injections into the right femoral condyles; there was no significant local bone loss. The structural continuity of the trabeculae was ensured, and the trabecular number and thickness did not significantly decrease. Compared to the Model + AAV and Model groups, the expression levels of the osteoblast activity-related genes *Alp*, *Bglap*, and *Col1α1* increased significantly, and those of the osteoclast activity-related genes *NFATc1*, *Acp5*, *Ctsk*, *Mmp9*, and *Clcn7* decreased significantly. These findings suggest that AAV-anti-miRNA-214 prevents osteoporosis via effective bidirectional regulation of bone metabolism, promoting osteoblasts but inhibiting osteoclast activity, reducing bone loss, and effectively delaying osteoporotic pathology.

We have created a new direction toward the early diagnosis, prevention, and treatment of osteoporosis. Molecular markers of early osteoporosis should be further screened and verified because they may aid diagnosis. It is also important to seek drugs that effectively balance osteogenesis and osteoclast function, improve the bone microenvironment, and (when given early) prevent osteoporosis occurrence/development. When treating patients, it is possible to target the sites prone to osteoporotic fractures. Local interventions will enhance the bone masses of the hip, distal radius, spine, and other sites, avoiding osteoporotic fractures caused by falls.

## Data Availability

The original contributions presented in the study are included in the article/supplementary material, further inquiries can be directed to the corresponding authors.
